# Supplementary material for: Two-dimensional gersiloxenes with tunable bandgap for photocatalytic H2 evolution and CO2 photoreduction to CO
Source: Nat Commun. 2020 Mar 19;11:1443. doi: 10.1038/s41467-020-15262-4 (PMC7081354; doi:10.1038/s41467-020-15262-4)
Supplement: Supplementary file 1 — Supplementary Information [file 41467_2020_15262_MOESM1_ESM.pdf]

Supplementary Information

Two-dimensional gersiloxenes with tunable bandgap for photocatalytic H_2 evolution and CO_2 photoreduction to CO

Zhao et al.

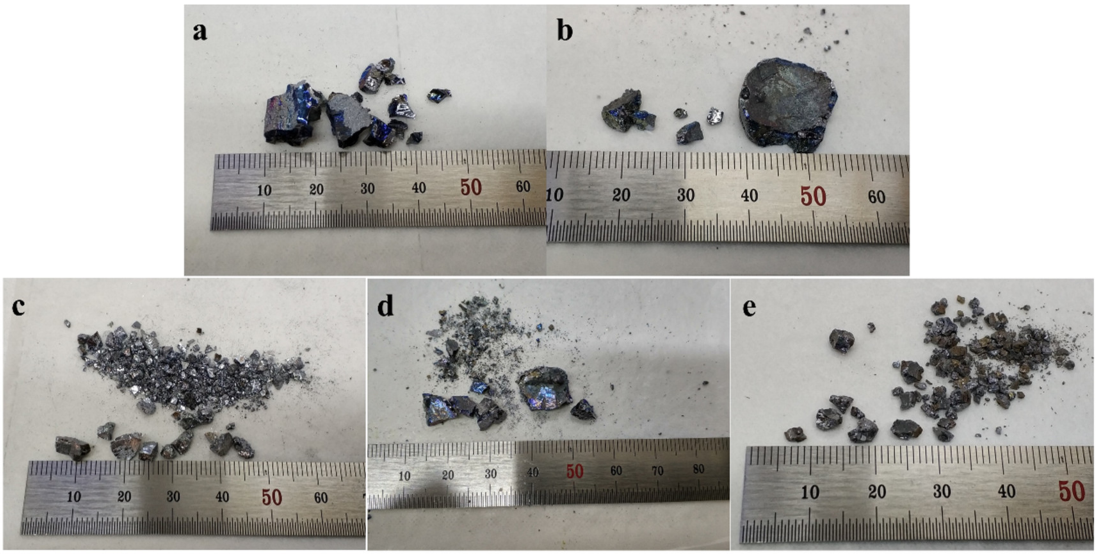

Supplementary Fig. 1. Optical images of $CaGe_{2(1-x)}Si_{2x}$ captured in an argon glove box. (a)–(e) x = 0.1, 0.3, 0.5, 0.7, 0.9.

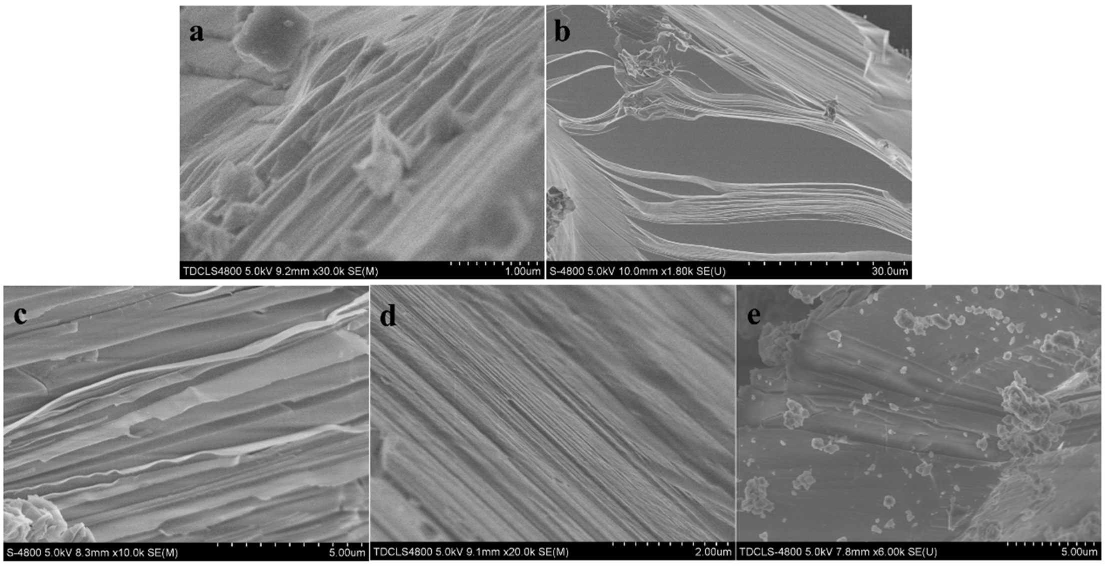

Supplementary Fig. 2. SEM images of $CaGe_{2(1-x)}Si_{2x}$. (a)–(e) x = 0.1, 0.3, 0.5, 0.7, 0.9.

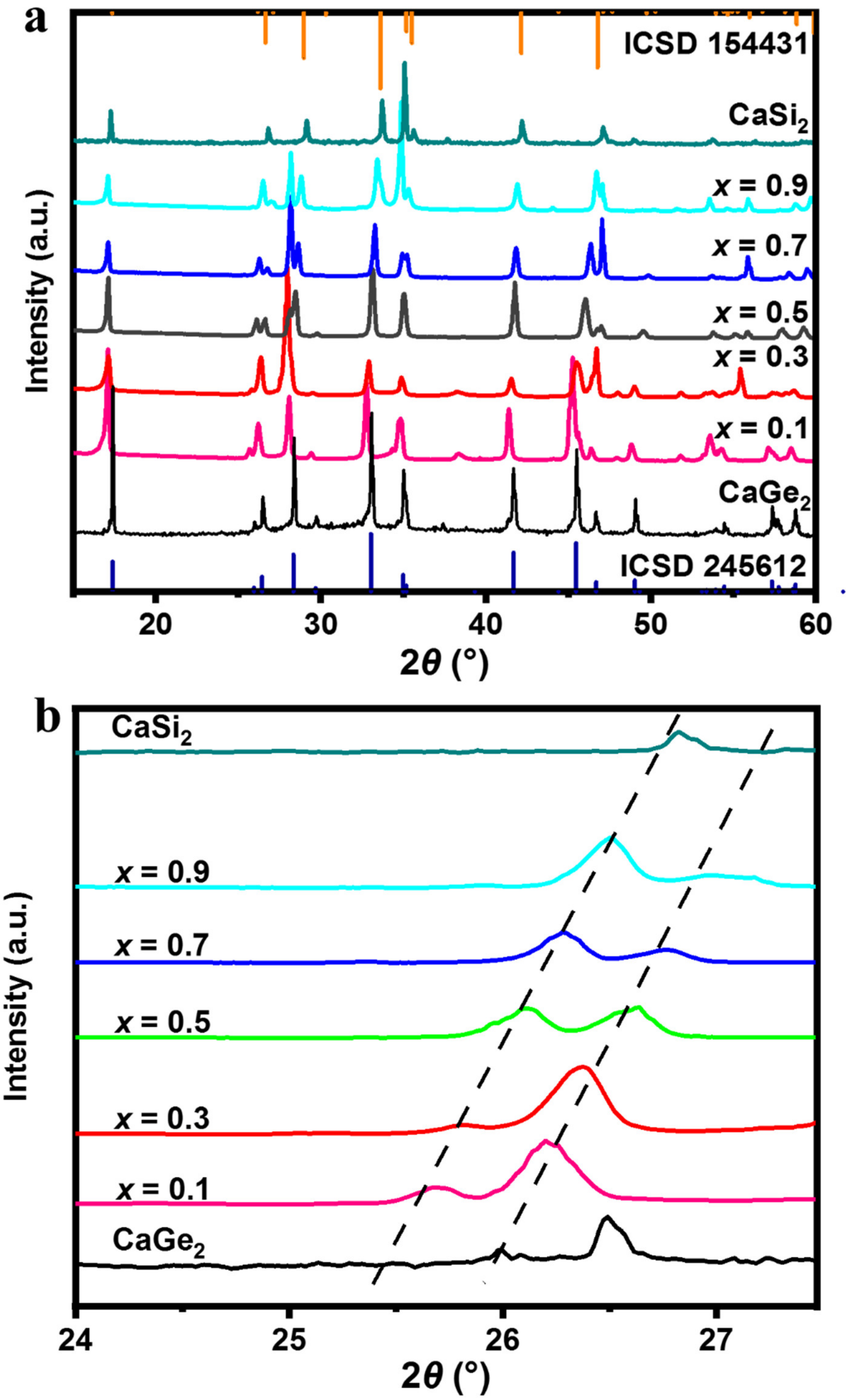

Supplementary Fig. 3. XRD patterns of the precursor $CaGe_{2-2x}Si_{2x}$ (x = 0.1, 0.3, 0.5, 0.7, 0.9), $CaGe_2$ and $CaSi_2$.The vertical lines at the top and bottom in (a) are the Inorganic Crystal Structure Database (ICSD) for $CaSi_2$ (ICSD154431) and $CaGe_2$ (ICSD245612). (b) The corresponding enlarged XRD patterns.

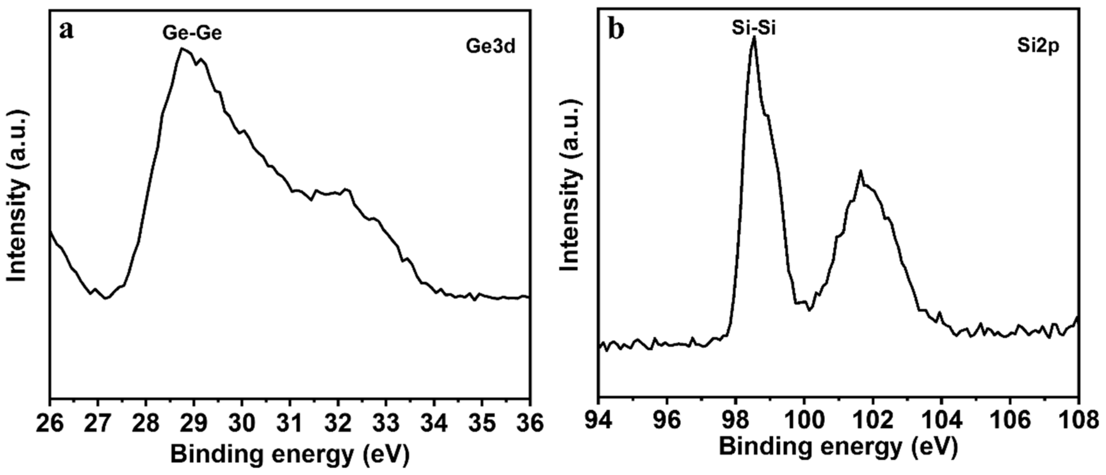

Supplementary Fig. 4. High-resolution XPS spectra of (a) Ge3d, and (b) Si2p for precursor $CaGe_{2-2x}Si_{2x}$, $x = 0.5$.

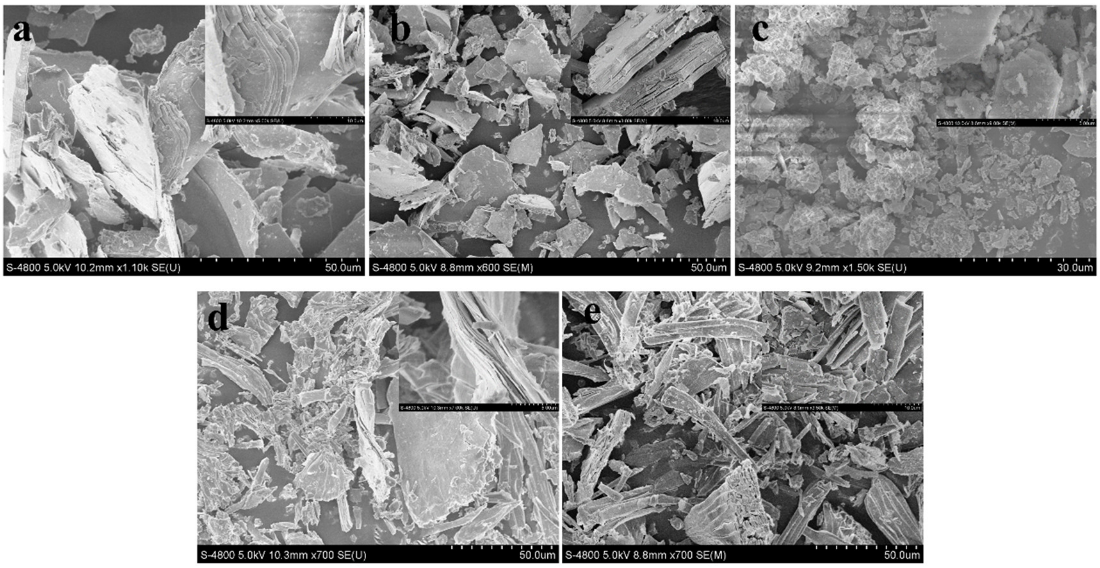

Supplementary Fig. 5. SEM images of 2D gersiloxenes with different x values. (a) $x = 0.1$, (b) $x = 0.3$, (c) $x = 0.5$, (d) $x = 0.7$, (e) $x = 0.9$.

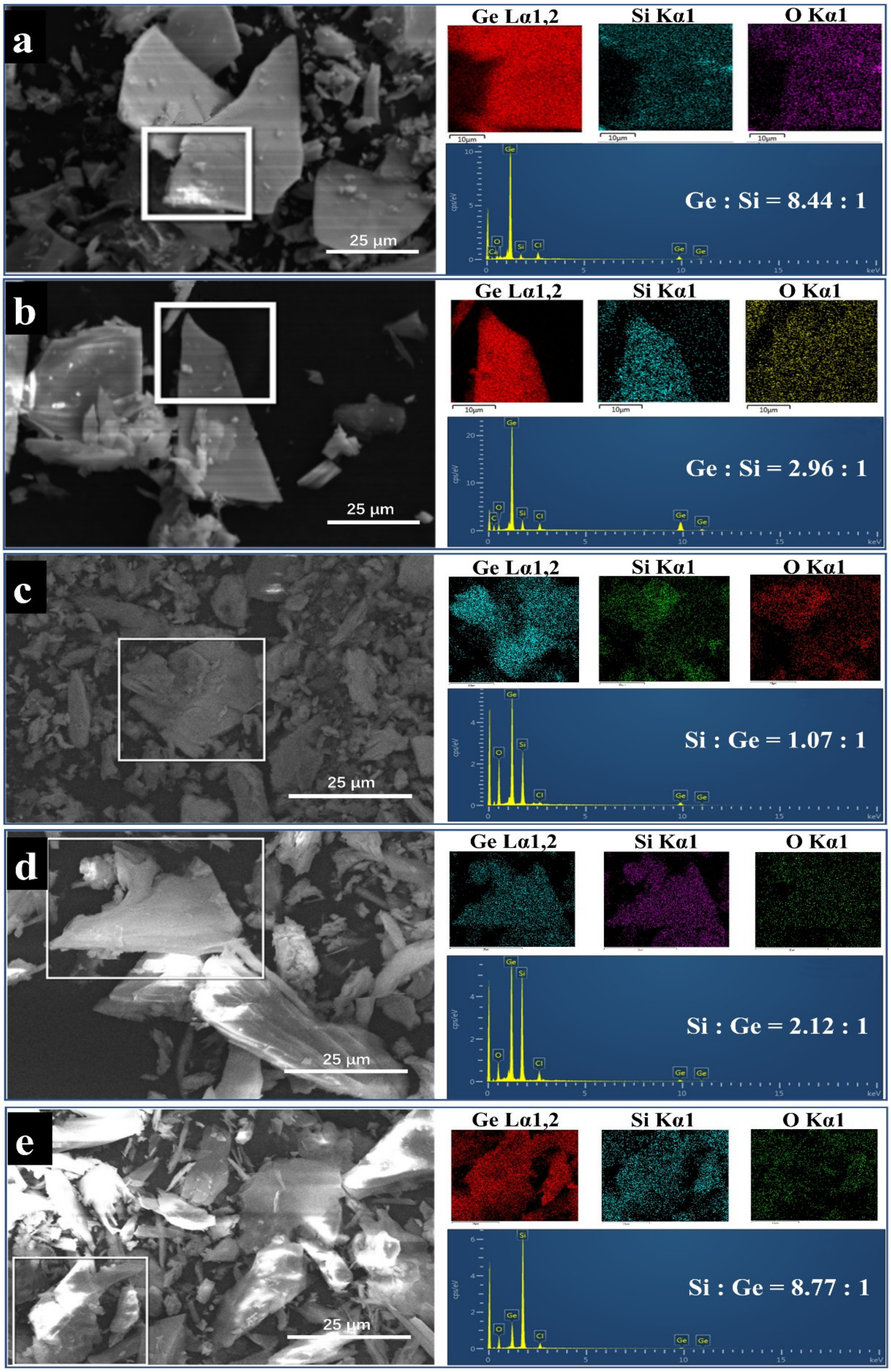

Supplementary Fig. 6. SEM elemental mapping and corresponding energy-dispersive spectroscopy (EDS) of gersiloxenes with x = 0.1–0.9 (a-e).

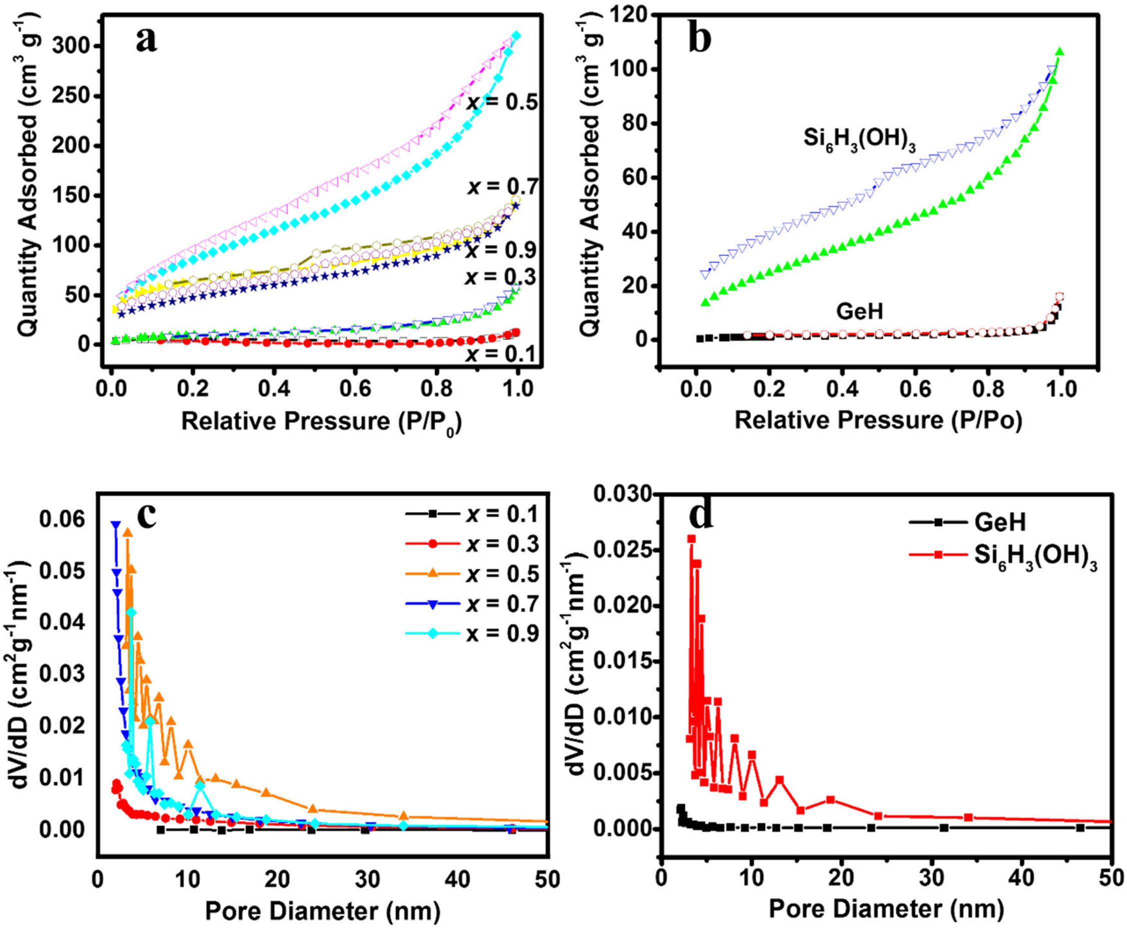

Supplementary Fig. 7. Adsorption (filled) and desorption (empty) isotherms of N_2 at 77 k for (a) gersiloxenes with $x = 0.1–0.9$. (b) GeH and $Si_6H_3(OH)_3$. Pore-size distributions of gersiloxenes with $x = 0.1–0.9$ (c), GeH and $Si_6H_3(OH)_3$ (d).

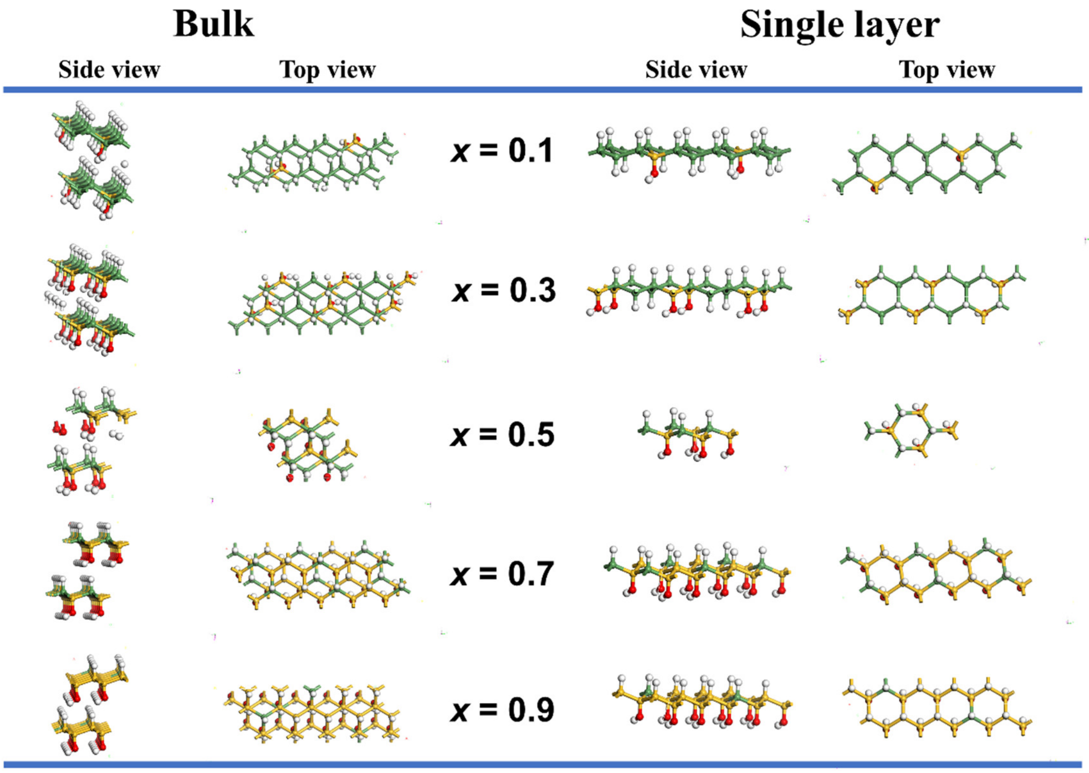

Supplementary Fig. 8. Isolated single layer and two-layer unit cell for monolayer and bulk 2D $Ge_{1-x}Si_xH_{1-y}(OH)_y$ alloys ($(GeH)_{1-x}(SiOH)_x$, $x < 0.5$, and $(GeH)_{1-x}Si_x(OH)_{0.5}H_{x-0.5}$, $x \geq 0.5$).

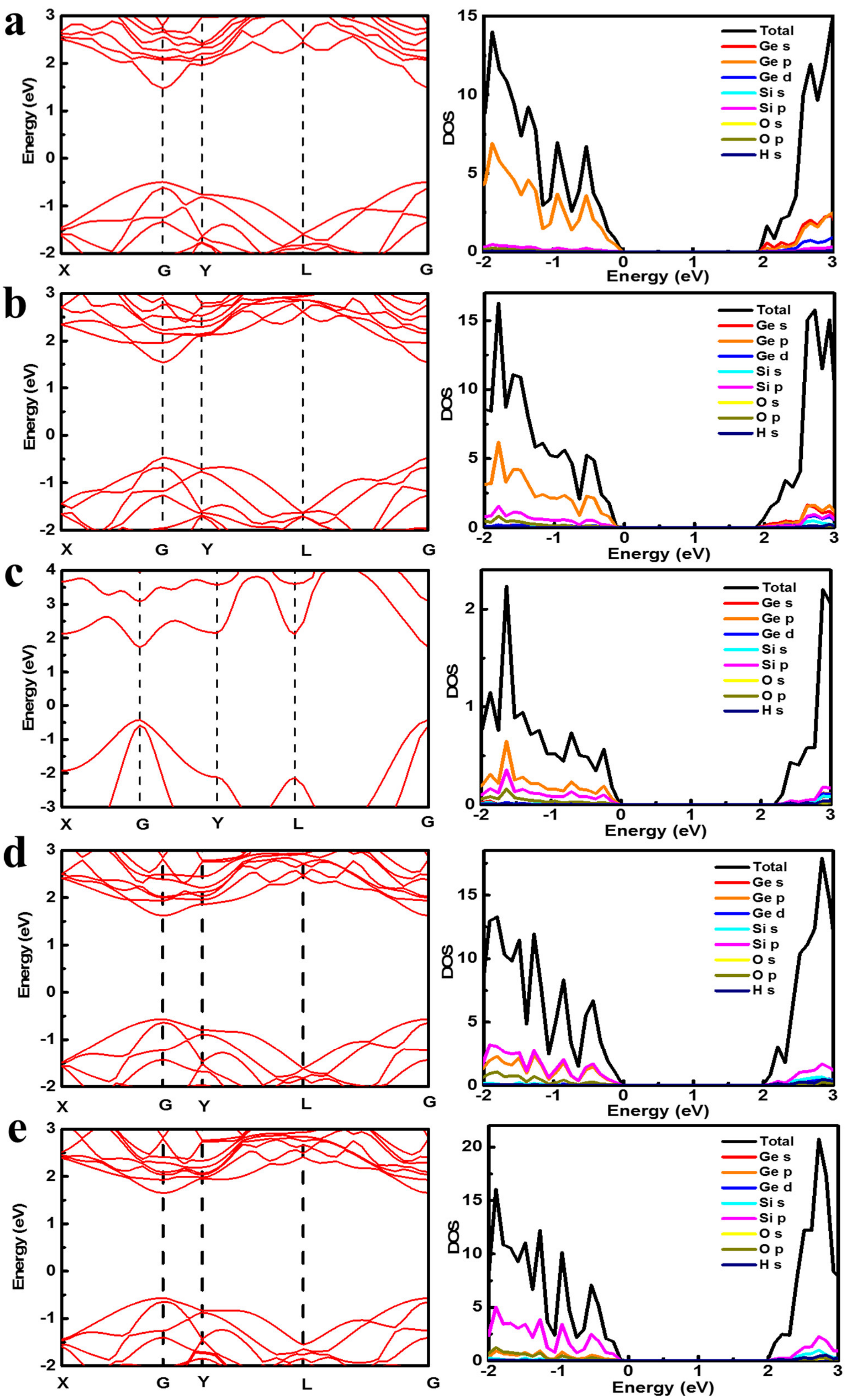

Supplementary Fig. 9. Electronic band structure and PDOS of single-layer unit cells for monolayer gersiloxenes with different x values. (a) $x = 0.1$, (b) $x = 0.3$, (c) $x = 0.5$, (d) $x = 0.7$, (e) $x = 0.9$.

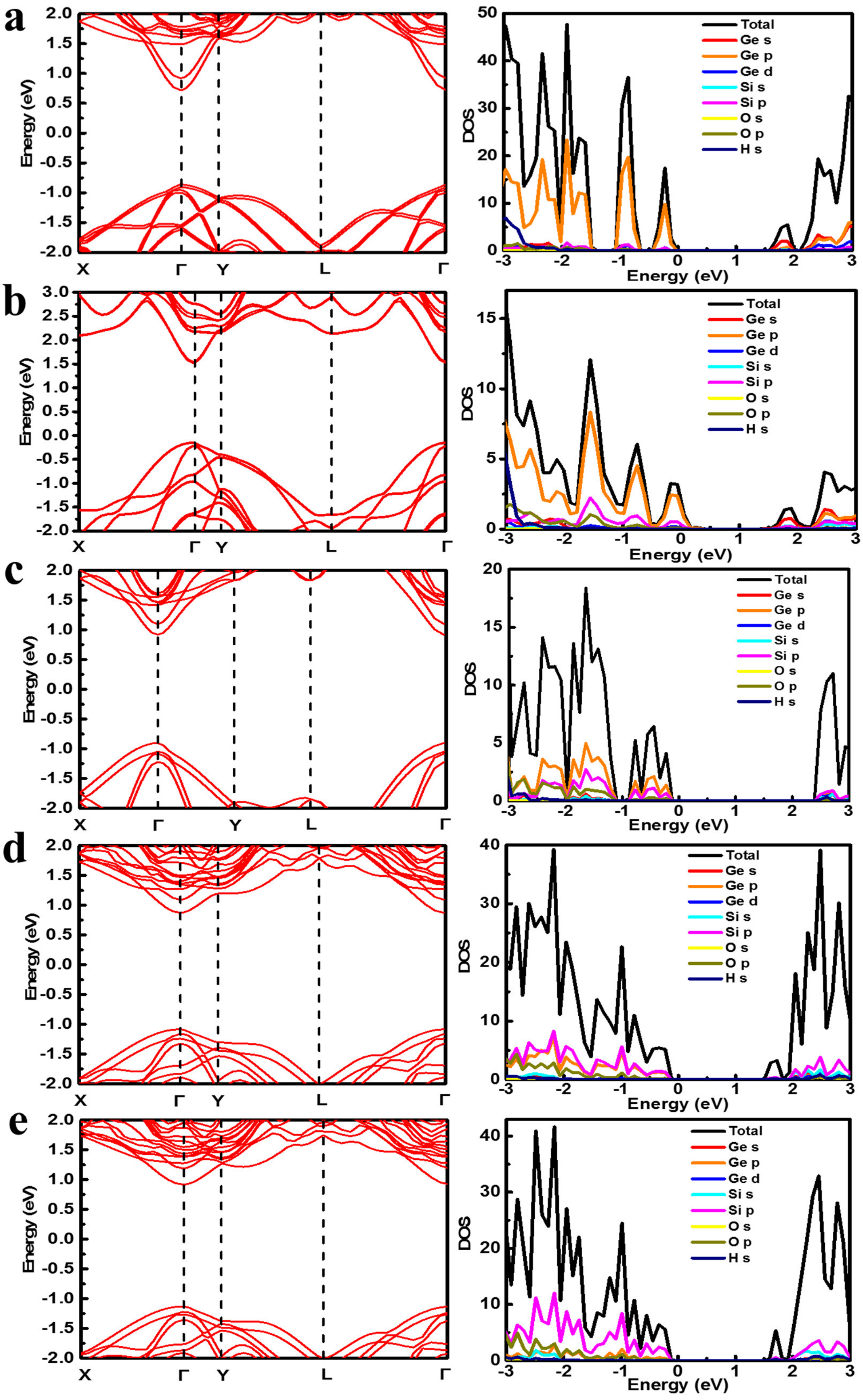

Supplementary Fig. 10. Electronic band structure and PDOS of two-layer unit cell for bulk gersiloxenes with different x values. (a) $x = 0.1$, (b) $x = 0.3$, (c) $x = 0.5$, (d) $x = 0.7$, (e) $x = 0.9$.

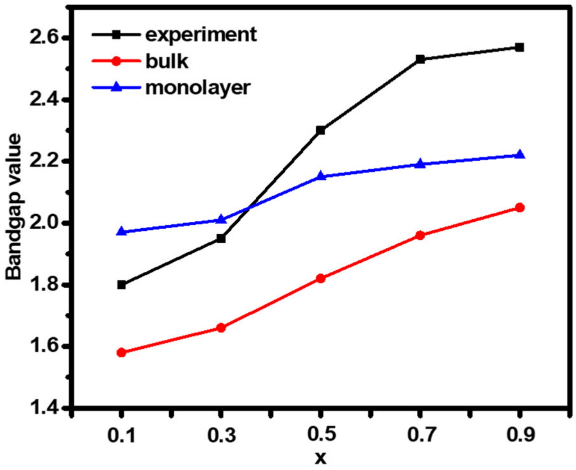

Supplementary Fig. 11. Evolution curves of band gap value depending on *x* for the experiment, theoretical calculation results with bulk and monolayer structures.

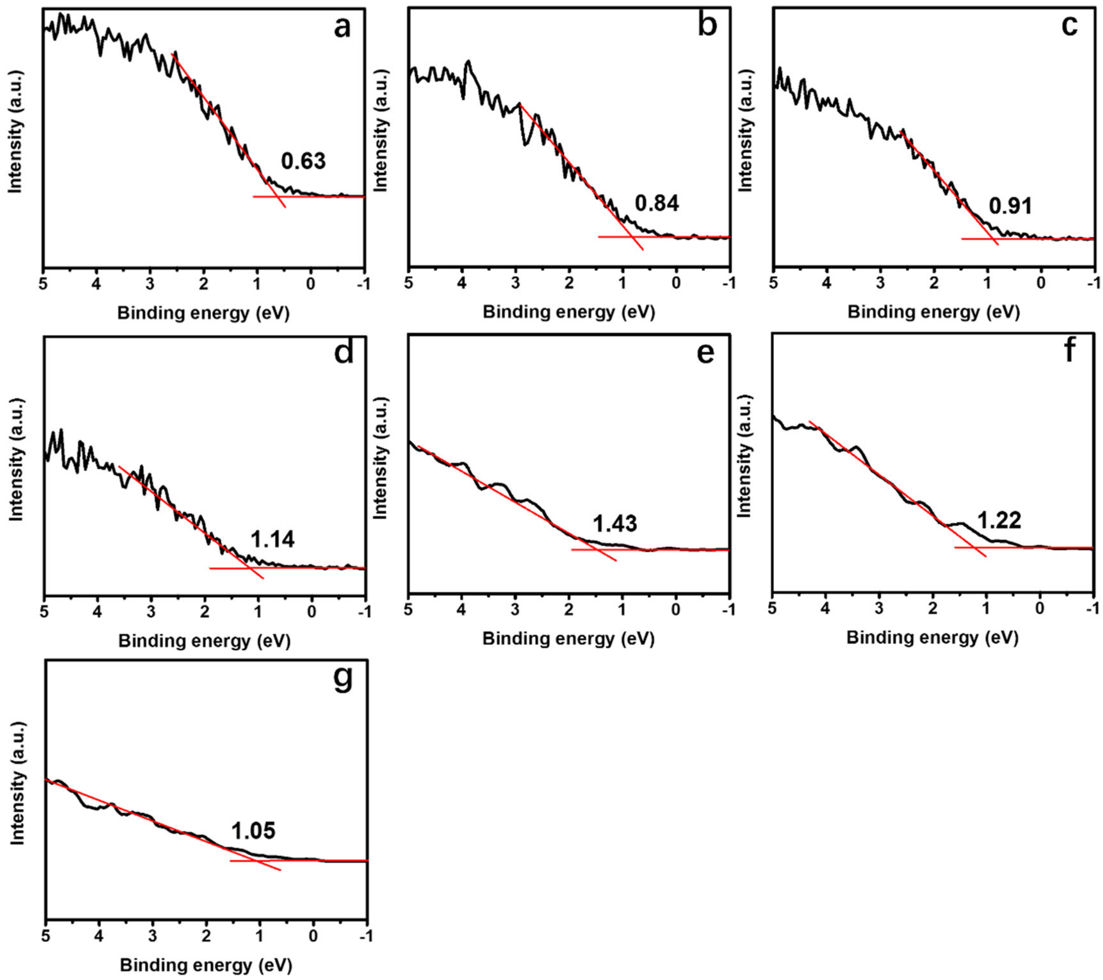

Supplementary Fig. 12. XPS valence band spectra of 2D gersiloxenes with different x value, GeH and $Si_6H_3(OH)_3$. (a) GeH, (b) x = 0.1, (c) x = 0.3, (d) x = 0.5, (e) x = 0.7, (f) x = 0.9, and (g) $Si_6H_3(OH)_3$.

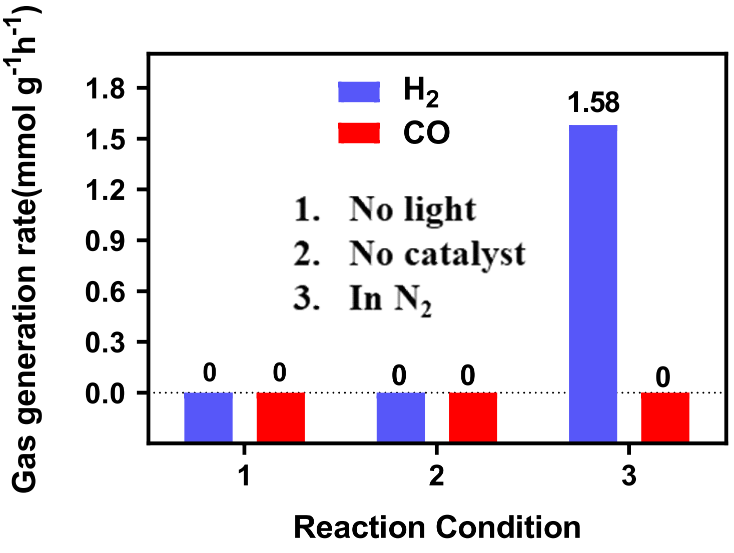

Supplementary Fig. 13. Photocatalytic CO_2 reduction rates of HGeSiOH with purging highly-pure N_2 instead of CO_2, no light, and no catalyst.

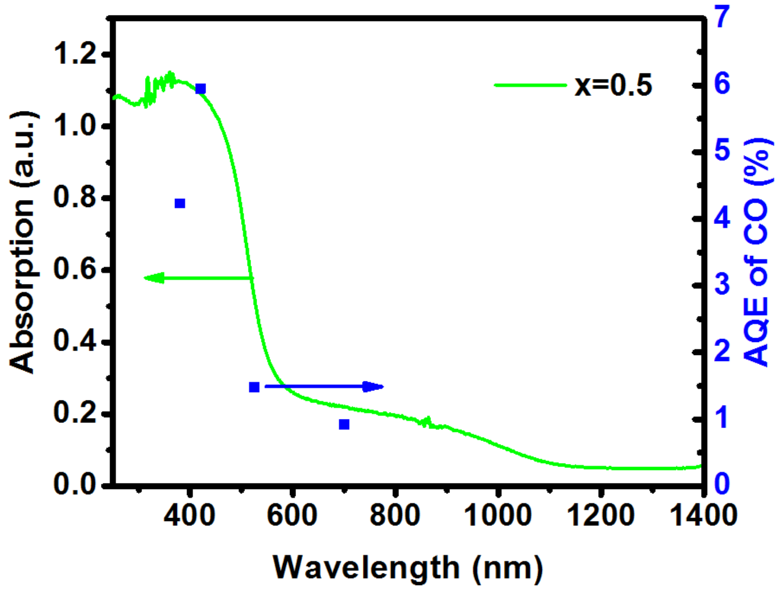

Supplementary Fig. 14. The action spectra and wavelength-dependent *AQE* of gersiloxene with x = 0.5 (HGeSiOH).

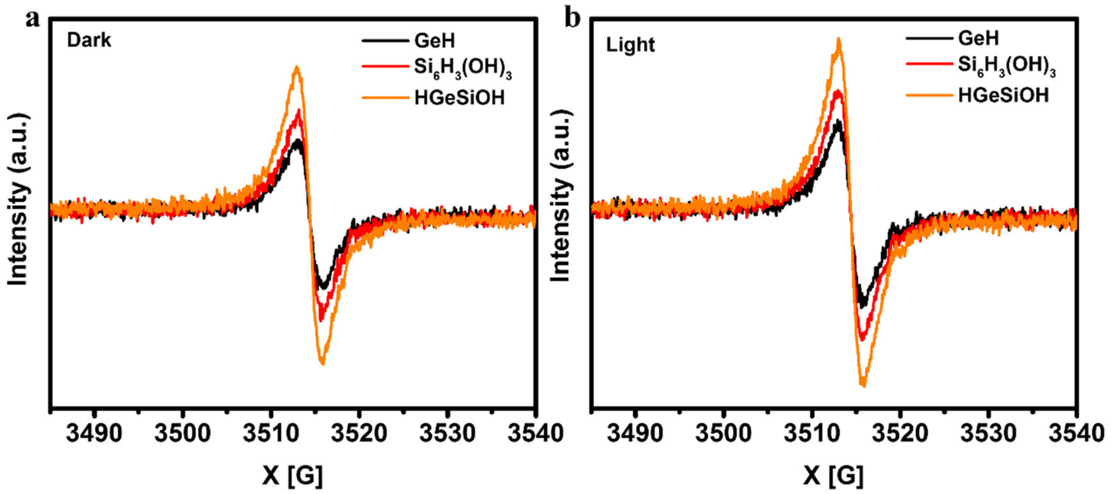

Supplementary Fig. 15. ESR spectra of GeH, HGeSiOH, and $Si_6H_3(OH)_3$ under dark (a) and light (b).

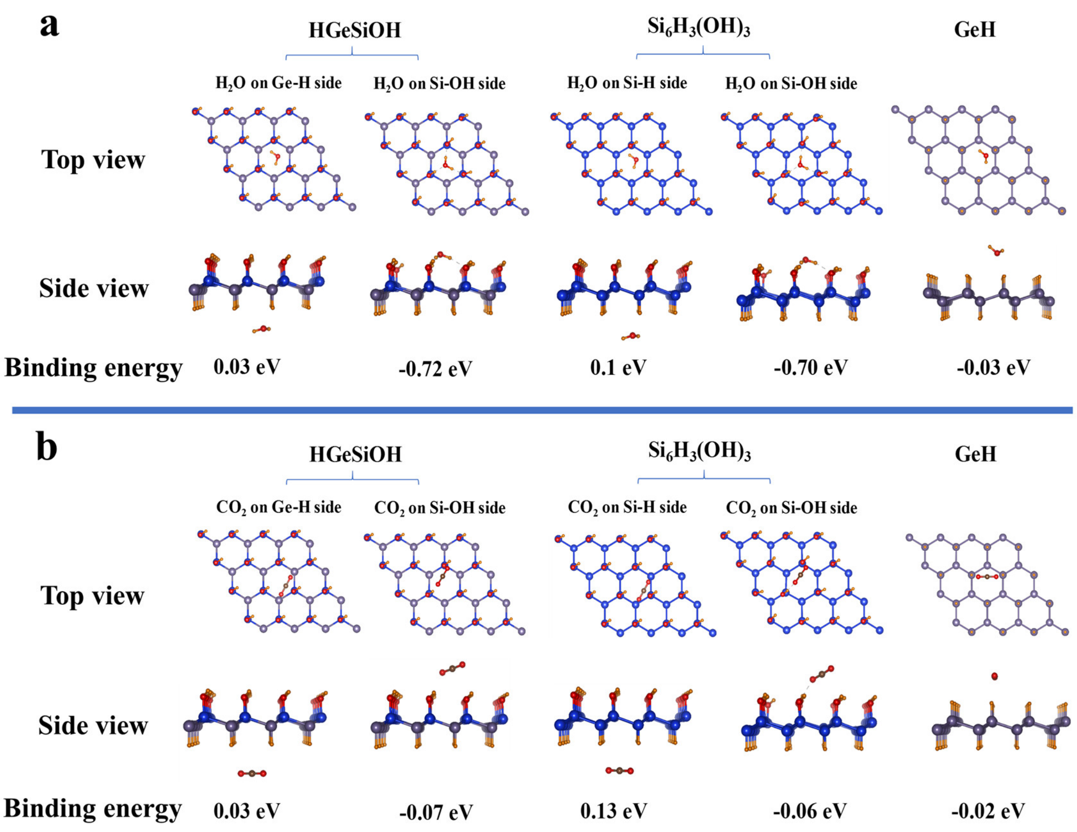

Supplementary Fig. 16. VASP optimized geometric structures and binding energies for H_2O (a) and CO_2 (b) adsorption on HGeSiOH, Si6H3(OH)3, and GeH monolayers.

Supplementary Table 1. The refined lattice parameters of as-prepared $CaGe_{2-2x}Si_{2x}$.

lattice parameters \ *x* value	0.1	0.3	0.5	0.7	0.9
a/b (Å)	3.9837(8)	3.9628(3)	3.9268(1)	3.8927(6)	3.8613(4)
c (Å)	30.6151(8)	30.5844(7)	30.5792(6)	30.5224(1)	30.5763(2)
Rwp	8.82%	8.78%	4.59%	6.19%	8.82%
Rp	5.76%	6.12%	2.99%	3.87%	5.95%

Supplementary Table 2. The calculated major orbital composition of the VB and CB of monolayer gersiloxenes with different *x* values.

x	0.1	0.3	0.5	0.7	0.9
**VB**	**Gep**, Ged, Sip	**Gep**, Sip, Ged, Ges	**Gep**, Op, Ged, Hs, Sis	**Sip**, Gep, Op, Ged	**Sip**, Gep, Op
**CB**	**Ges**, Gep, Ged, Sip, Sis	**Ges**, Gep, Sip, Sis, Ged, Hs, Os	**Sip**, Gep, Ged, Hs, Sis, Os, Ges	**Sip**, Hs, Sis, Os, Ged, Gep	**Sip**, Hs, Sis, Os, Op, Ged, Gep

The underlined orbital is the orbital with the highest proportion.

Supplementary Table 3. The calculated major composition orbits of VB and CB of bulk gersiloxenes with different *x* values.

x	0.1	0.3	0.5	0.7	0.9
**VB**	**Gep**, Ged, Sis	**Gep**, Sip	**Gep,** Ged, Sis, Sip	**Sip**, Gep	**Sip**, Ges, Gep
**CB**	**Ges**, Gep	**Ges**, Gep, Sis, Sip	Sis, **Sip,** Ges, Gep, Ged	**Sis**, Sip, Hs	Sis, **Sip**, Hs, Os

The underlined orbital is the orbital with the highest proportion.

Supplementary Table 4. The data of *AQE* under incident light with different wavelengths.

**Wavelength (nm)**	**CO production amount (mmol)**	**P (10^{-3} W/cm^2)**	***AQE* (%)**
**380**	7.780	0.41	4.23
**420**	10.624	0.36	5.95
**525**	3.492	0.38	1.48
**700**	1.450	0.19	0.92

Supplementary Table 5. Comparison with other high-performance photocatalytic systems for CO_2 reduction to CO.

Photocatalyst	Catalyst amount, Reaction solution	Light source, reaction temperature (℃)	COER (μmol $g^{-1}h^{-1}$)	*AQE*	References
CPOP-30-Re	10 mg, 10−20 vol % TEOA or TEA in DMF or CH_3CN	300 W xenon (λ > 390 nm), 20 °C	623	—	1
$BiOBr_{0.6}Cl_{0.4}$	10 mg, 20 μL of pure water	300 W xenon lamp, 298 K	15.86	—	2
$InVO_4$	0.1 g, 0.4 ml of deionized water, CO_2 gas	300 W xenon lamp, room temperature	18.28	—	3
Ni-TpBpy	0.01 mmol of $[Ru(bpy)_3]Cl_2·6H_2O$ were carried out in 30 μL CH_3CN and 10 μL H_2O)	300 W xenon lamp (λ ≥ 420 nm), 298 K.	811.4	0.3% at 420 nm	4
$Sr_2Bi_2Nb_2TiO_{12}$	0.1 g, 1.3 g $NaHCO_3$ + 5 mL H_2SO_4 (4 M)	300 W Xe lamp 20 ℃	17.11	—	5
PEosinY-1	10 mg, 10 mL of aqueous solution	300 W Xe lamp (λ ≥ 420 nm), 25 ℃	33	—	6
$SrNb_2O_6$	10 mg, 4 mL water	Xe lamp (λ = 300−780 nm), 323 K	16.6	—	7
MOF-525-Co	2 mg, MeCN/TEOA	300 W xenon arc lamp (400−800 nm)	CO (200.6), CH_4 (36.67)		8

$ZnIn_2S_4$	0.1 g, 2 mL deionized water	300 W Xe lamp with a standard AM 1.5 filter, 298 ± 0.2 K	33.2	0.23% at 400 nm	9
TiO_2/N-doped-RGO	10 mg, water bubbler	400W Xenon lamp (250–400 nm)	50	—	10
N-GQDs/Vo-NaTaON	20 mg, 5 mL distilled water CO_2/H_2 gas	300 W Xe lamp (λ > 400 nm)	CO (43), CH_4 (10)	—	11
Co-ZIF-9/TiO_2	50 mg, 3 mL of deionized water	300 W Xe lamp (200–900 nm)	CO (17.58), CH_4 (1.98), H_2 (2.6)	—	12
HCP-TiO_2-FG	20 mg, (gas–solid reaction conditions)	300 W Xe lamp (λ > 420 nm), about 50 °C	CO (21.63), CH_4 (27.62)	—	13
	CO_2 gas	UV-vis, about 50 °C	CO (39.51), CH_4 (51.23),	—	
MOF-Ni	5 mg, $[Ru(bpy)_3]Cl_2.6H_2O$ (bpy= 2,2'-bipyridine, 0.01 mmol), acetonitrile (26 mL), H_2O (2 mL), and TIPA (2 mL).	300 W Xe lamp (420–800 nm), 303 K	CO (371.6), H_2 (8.3)	5.3×10^{-3} % at 420 nm	14
MOF-Co			CO (1140.0), H_2 (1265.0)		
MOF-Cu			CO (68.0), H_2 (232.0)		
Pt/NaTaO3	0.07 g, 3 mL of distilled water, CO_2 and H_2	UV Xe lamp, 300 W (λ > 200nm)	139.1	—	15
ncSi:H	several mg,	Xe lamp (~ 15 suns),	250	—	16

	H_2 and CO_2 1:1 (gas phase reaction)	150 °C			
Pd@SiNS	10 mg, CO_2 and H_2 at a 1:1 ratio (gas phase reaction)	300 W Xe lamp (~ 15 suns), 170 °C	10	—	17
CdS-CoE-350	25 mg, 70 mL of Na_2CO_3 and Na_2SO_3 solution (0.3 mol/L and 0.06 mol/L)	300 W Xe lamp (λ > 420nm),	392	2.2% at 420 nm	18
Pt/o-PCN	0.01 g catalysts were mixed with 40 mL $KHCO_3$ (0.1 M), Na_2S (0.1 M) and Na_2SO_3 (0.1 M) aqueous solution	300 W Xe lamp (λ > 400 nm), room temperature	286	3.337% at 420 nm	19
HGeSiOH	60 mg, 50 ml sodium sulfite solution (Na_2SO_3, 0.10 M), CO_2 gas	300 W Xe lamp, 25°C	6910	5.95% at 420 nm	This work

Supplementary Note 1

Morphology, structure and chemical characterization of the CaGe2-2xSi2x.

Zintl-phase $CaSi_2$, $CaGe_2$, and $Ca(Ge_{1-x}Si_x)_2$ ($0 < x < 1$) have already been reported to be synthesized by epitaxial growth[20-22]. However, these materials are limited by their high cost and are difficult to prepare in large quantities. In recent years, $CaSi_2$ and $CaGe_2$ have been synthesized by directly annealing elemental calcium with silicon and germanium under vacuum conditions[23,24]. Therefore, it is reasonable to believe that $Ca(Ge_{1-x}Si_x)_2$ can also be prepared in a similar way. By annealing stoichiometric amounts of calcium, germanium and silicon, we obtained $CaGe_{2-2x}Si_{2x}$ with $x = 0.1$, 0.3, 0.5, 0.7, and 0.9. All the products are bright black crystals with metallic luster (Supplementary Fig. 1). Scanning electron microscopy (SEM) images (Supplementary Fig. 2) show stacking lamellar morphology for all compositions, and the sample with $x = 0.5$ exhibits the most striking layer-by-layer feature. X-ray diffraction (XRD) patterns (Supplementary Fig. 3a) show no evidence for phase separation of the alloyed Zintl phases into the pure phases $CaSi_2$ and $CaGe_2$, and the as-prepared $CaGe_{2-2x}Si_{2x}$ crystals exhibit the same trigonal rhombohedral tr6 crystal structure as $CaSi_2$ and $CaGe_2$[25]. The enlarged XRD patterns in the range of 24–27° (Supplementary Fig. 3b) show that as the ratio of Si increases to replace Ge in $CaGe_{2-2x}Si_{2x}$ solid solutions, the diffraction peaks at about 25.99° and 26.48° corresponding to (101) and (012) facets gradually shift toward higher 2θ angles, owing to the smaller radius of Si with respect to Ge for shrinking atomic distance[26]. This further confirmed the successful synthesis of the random alloys of $CaGe_{2-2x}Si_{2x}$.

Moreover, the lattice constant a gradually changes from 3.9837 to 3.8613 Å as x increases from 0.1 to 0.9 (shown in Supplementary Table 1), following the Vegard's law, which is consistent with that of the epitaxial Zintl-phase calcium germanosilicide alloy films[20].

Supplementary Note 2

Brunauer–Emmett–Teller (BET) surface area analysis. The N_2 Adsorption/desorption isotherms (Supplementary Fig. 7a and b) exhibit a type-IV isotherm with hysteretic loops, implying the mesoporous structure of these materials. The pore-size distribution curves (Supplementary Fig. 7c and d) show that the pore sizes for all gersiloxenes, GeH, and $Si_6H_3(OH)_3$ lie in the range of the mesopore. For all samples, gersiloxene with $x = 0.5$ exhibits the most extensive size distribution. These pores are mainly composed of interlayer voids of nanosheets and stacked gaps between adjacent nanosheets.

Supplementary Note 3

Structural models, band structures, VBMs and CBMs of gersiloxenes. Based on the experimental results, we established a series of structural models (shown in Supplementary Fig. 8) of isolated single-layer and two-layer unit cells for monolayer and bulk 2D $Ge_{1-x}Si_xH_{1-y}(OH)_y$ alloys ($(GeH)_{1-x}(SiOH)_x$, $x < 0.5$, and $(GeH)_{1-x}Si_x(OH)_{0.5}H_{x-0.5}$, $x \geq 0.5$) to investigate the electronic band structure and partial density of states (PDOS) using periodic density-functional theory (DFT) calculations with the Heyd−Scuseria−Ernzerhof hybrid functional (HSE06). Supplementary Fig. 9 shows the band structure for monolayer $(GeH)_{1-x}(SiOH)_x$ ($x < 0.5$) and $(GeH)_{1-x}Si_x(OH)_{0.5}H_{x-0.5}$ ($x \geq 0.5$), from which we know all the samples with x = 0.1–0.9 are direct-bandgap semiconductors with both the conduction band minimum (CBM) and valence band maximum (VBM) occurring at the G point. The calculated bandgaps for gersiloxenes with x = 0.1, 0.3, 0.5, 0.7, and 0.9 are 1.97, 2.01, 2.15, 2.19, and 2.22 eV, respectively. In addition, bulk structures show the same bandgap type as monolayer structures; that is, they are all direct bandgap semiconductors, and the bandgap values are 1.58, 1.66, 1.82, 1.96, and 2.05 eV, respectively (Supplementary Fig. 10), which increase with the value of x. These results are in good agreement with the experimental bandgap values. (Considering the existence of Ge–Cl, Si–Cl generated during the topotactic deintercalation process [27,28,29]. However, from the XPS results, we obtained that the chlorine in the molar ratio Cl/(Ge + Si) are 0.05, 0.04, 0.06, 0.04, and 0.05 for gersiloxenes with x = 0.1, 0.3, 0.5, 0.7, and 0.9, respectively. The practice content of Cl atoms in our synthesized gersiloxene nanosheets are very low, so the influence on the

electronic properties of the gersiloxenes is negligible. Therefore, it is reasonable that there are some errors between the theoretical results and the experimental values.) Since the gersiloxene nanosheets obtained in our experiment are prepared from the liquid phase dispersion, which are few-layer stacking structure with a thickness of about 3–6 nm. As is known to all, 2D materials have a trend of re-stacking after the liquid phase exfoliation into monolayer nanosheets. Therefore, the calculated results help us to judge whether the properties of the obtained few-layer nanosheets are closer to that of the single-layer structure or the bulk structure. It can help us understand theoretical calculations and experimental results. Obviously, the electronic properties of the experimentally obtained few-layer nanosheets are more in conformity with the single-layer structure, rather than the bulk structure that is tightly packed by the layers under the van der Waals force (Supplementary Fig. 11). The band structures (Supplementary Fig. 9 and Fig. 10) show that the CBM or VBM of all gersiloxenes are strongly dispersed, indicating a very low effective mass for photogenerated carriers. The PDOS results suggest that the electronic states of the CB and VB near the Fermi level are hybridized by different orbitals of Ge and Si, O, and H (as shown in Supplementary Table 2 and Table 3). Thus, the separation and transportation of electrons and holes would be affected by the spatial charge distribution of the CBM and VBM[30,31]. The strong dispersion of the energy level structure is beneficial for the migration of photogenerated electrons and can suppress the recombination of photogenerated electrons and holes. Notably, the VBs for samples with $x = 0.1$, 0.3, and 0.5 are dominated by

Ge p-orbitals, while for x = 0.7 and 0.9, the VBs are dominated by Si p-orbitals. On the other hand, the CBs for samples with x = 0.1, 0.3, 0.5, 0.7, and 0.9 mainly originate from Ge s-orbitals, Ge s-orbitals, Si p-orbitals, Si p-orbitals, and Si p-orbitals, respectively. That is, for gersiloxene with x = 0.5, i.e., HGeSiOH, the distribution of the VBM and CBM is similar to that of type-II heterostructures[32-36], and the photoinduced electrons and holes would transfer to the CBM (Si) and VBM (Ge), respectively, which is more conducive to the separation of excited electrons and holes.

Supplementary Note 4

Comparison of CO_2 photoreduction under different conditions. Comparison tests were performed to confirm the photocatalysis ability of the synthesized catalyst. The results are shown in Supplementary Fig. 13. To exclude the possibility of influence of organic impurities on the sample surface, highly-pure N_2 is purged into the reactor instead of CO_2. No CO can be detected during the photoreaction process in N_2 which proves that CO is not produced by the possible organic impurities on the gersiloxene surface. Moreover, no CO was detected with no light irradiation or without gersiloxene catalyst, further confirming that it is the gersiloxene catalyst initiating the reaction.

Supplementary Note 5

***AQE* results.** To further confirm the activity of photocatalytic CO_2 reduction, we performed photocatalytic CO_2 reduction experiments under the monochromatic light irradiations with different wavelength to calculate the apparent quantum efficiency. The test conditions are the same except for the irradiations. The photocatalytic reduction of CO_2 to CO activities of gersiloxene with $x = 0.5$ (HGeSiOH) under monochromatic irradiations with wavelengths of 380, 420, 525 and 700 nm are listed in Supplementary Table 4, and the action spectra and wavelength-dependent *AQE* are shown in Supplementary Fig. 14. The calculated highest *AQE* is 5.95% at 420 nm.

Supplementary Note 6

Discussion on the adsorption energy calculated by VASP. In order to better understand the influence of structure on adsorption energy, we also used VASP to calculate the adsorption energy for comparison which material researchers often do. As shown in Supplementary Fig. 16a and b, the H_2O or CO_2 adsorption energy on HGeSiOH is more negative than that on $Si_6H_3(OH)_3$ and GeH, suggesting that the alloyed structure of 2D GeSi can significantly improve its H_2O or CO_2 adsorption capacity. The difference from the result of Materials Studio calculation is the VASP calculations demonstrated that the interactions of an H_2O or CO_2 molecule with the Ge–H and Si–H of HGeSiOH and $Si_6H_3(OH)_3$ are not energetically favorable, which indicates that Si–OH in HGeSiOH and $Si_6H_3(OH)_3$ has a strong adsorption effect on H_2O and CO_2, which makes the adsorption of Ge–H for H_2O and CO_2 unstable. Therefore, it can be concluded that both H_2O and CO_2 may be adsorbed and activated at the –OH sites.

Supplementary References

1. Liang, H., et al. Rhenium-Metalated Polypyridine-Based Porous Polycarbazoles for Visible Light CO_2 Photoreduction. *ACS Catal.* **9**, 3959-3968 (2019).
2. Gao, M., et al. Persian buttercup-like $BiOBr_xCl_{1-x}$ solid solution for photocatalytic overall CO_2 reduction to CO and O_2. *Appl. Catal. B Environ.* **243**, 734-740 (2019).
3. Han, Q., et al. Convincing Synthesis of Atomically Thin, Single-Crystalline $InVO_4$ Sheets toward Promoting Highly Selective and Efficient Solar Conversion of CO_2 into CO. *J. Am. Chem. Soc.* **141**, 4209-4213 (2019).
4. Zhong, W., et al. A Covalent Organic Framework Bearing Single Ni Sites as a Synergistic Photocatalyst for Selective Photoreduction of CO_2 to CO. *J. Am. Chem. Soc.* **141**, 7615−7621 (2019).
5. Yu, H., et al. Three‐in‐One Oxygen Vacancies: Whole Visible−Spectrum Absorption, Efficient Charge Separation, and Surface Site Activation for Robust CO_2 Photoreduction. *Angew. Chem. Int. Ed. Engl.* **58**, 3880-3884 (2019).
6. Yu, X., et al. Eosin Y‐Functionalized Conjugated Organic Polymers for Visible‐Light‐Driven CO_2 Reduction with H_2O to CO with High Efficiency. *Angew. Chem. Int. Ed. Engl.* **58**, 632-636 (2019).
7. Xie, S., et al. $SrNb_2O_6$ nanoplates as efficient photocatalysts for the preferential reduction of CO_2 in the presence of H_2O. *Chem. Commun. (Camb.)* **51**, 3430-3433 (2015).
8. Zhang, H., et al. Efficient Visible‐Light‐Driven Carbon Dioxide Reduction by a Single‐Atom Implanted Metal–Organic Framework. *Angew. Chem. Int. Ed. Engl.*

55, 14310-14314 (2016).

9. Jiao, X., et al. Defect-mediated electron–hole separation in one-unit-cell $ZnIn_2S_4$ layers for boosted solar-driven CO_2 reduction. *J. Am. Chem. Soc.* **139**, 7586-7594 (2017).

10. Lin, L., et al. N-doped reduced graphene oxide promoted nano TiO_2 as a bifunctional adsorbent/photocatalyst for CO_2 photoreduction: Effect of N species. *Chem. Eng. J.* **316**, 449-460 (2017).

11. Hou, J., et al. Perovskite-based nanocubes with simultaneously improved visible-light absorption and charge separation enabling efficient photocatalytic CO_2 reduction. *Nano Energy* **30**, 59-68 (2016).

12. Yan, S., et al. Co-ZIF-9/TiO_2 nanostructure for superior CO_2 photoreduction activity. *J. Mater. Chem. A* **4**,15126-15133 (2016).

13. Wang, S., et al. Porous hypercrosslinked polymer-TiO_2-graphene composite photocatalysts for visible-light-driven CO_2 conversion. *Nat. Commun.* **10**, 676 (2019).

14. Wang, X., et al. Monometallic Catalytic Models Hosted in Stable Metal–Organic Frameworks for Tunable CO_2 Photoreduction. *ACS Catal.* **9**, 1726-1732 (2019).

15. Li, M., et al. Highly efficient and stable photocatalytic reduction of CO_2 to CH_4 over Ru loaded $NaTaO_3$. *Chem. Commun. (Camb.)* **51**, 7645-7648 (2015).

16. Sun, W., et al. Heterogeneous reduction of carbon dioxide by hydride-terminated silicon nanocrystals. *Nat. Commun.* **7**, 12553 (2016).

17. Qian, C., et al. Catalytic CO_2 reduction by palladium-decorated silicon–hydride

nanosheets. *Nat. Catal.* **2**, 46-54 (2019).

18. Zhao, G., et al. Efficient photocatalytic CO_2 reduction over Co (II) species modified CdS in aqueous solution. *Appl. Catal. B Environ.* **226**, 252-257 (2018).

19. Li, A., et al. Three−Phase Photocatalysis for the Enhanced Selectivity and Activity of CO_2 Reduction on Hydrophobic Surface. *Angew. Chem. Int. Ed.* **58**, 14549–14555 (2019).

20. Vogg, G., Miesner, C., Brandt, M. S., Stutzmann, M. & Abstreiter, G. Epitaxial alloy films of zintl-phase $Ca(Si_{1-x}Ge_x)_2$. *J. Cryst. Growth* **223**, 573-576 (2001).

21. Morar, J. & Wittmer, M. Growth of epitaxial $CaSi_2$ films on Si(111). *J. Vac. Sci. Technol. A* **6**, 1340-1342 (1988).

22. Vogg, G., et al. Epitaxial $CaGe_2$ films on germanium. *J. Cryst. Growth* **212**, 148-154 (2000).

23. Bianco, E., et al. Stability and exfoliation of germanane: a germanium graphane analogue. *ACS Nano* **7**, 4414-4421 (2013).

24. Yaokawa, R., Nakano, H. & Ohashi, M. Growth of $CaSi_2$ single-phase polycrystalline ingots using the phase relationship between $CaSi_2$ and associated phases. *Acta Mater.* **81**, 41-49 (2014).

25. Cultrara, N. D., et al. Synthesis of 1T, 2H, and 6R germanane polytypes. *Chem. Mater.* **30**, 1335-1343 (2018).

26. Gao, M..Yang, J.. Sun, T.. Zhang, Z.. Zhang, D.. Huang, H.. Lin, H.. Fang, Y.. Wang, X. Persian buttercup-like $BiOBr_xCl_{1-x}$ solid solution for photocatalytic overall CO_2 reduction to CO and O_2. *Appl. Catal. B Environ.* **243**, 734-740 (2019).

27. Bianco, E., et al. Stability and exfoliation of germanane: a germanium graphane analogue. *ACS Nano* **7,** 4414-4421 (2013).
28. Yamanaka, S., Matsu-Ura, H. & Ishikawa, M. New deintercalation reaction of calcium from calcium disilicide. Synthesis of layered polysilane. *Mater. Res. Bull.* **31**, 307-316 (1996).
29. Okamoto, H., et al. Silicon nanosheets and their self-assembled regular stacking structure. *J. Am. Chem. Soc.* **132**, 2710-2718 (2010).
30. Lv, X., et al. Two-dimensional germanium monochalcogenides for photocatalytic water splitting with high carrier mobility. *Appl. Catal. B Environ.* **217,** 275-284 (2017).
31. Zhang, H., et al. Computational studies on the structural, electronic and optical properties of graphene-like MXenes (M_2CT_2, M = Ti, Zr, Hf; T = O, F, OH) and their potential applications as visible-light driven photocatalysts. *J. Mater. Chem. A* **4,** 12913-12920 (2016).
32. Lv, X., Wei, W., Sun, Q., Li, F. Huang, B.; Dai, Y., Two-dimensional germanium monochalcogenides for photocatalytic water splitting with high carrier mobility. *Appl. Catal. B Environ.* **217**, 275-284 (2017).
33. Zhang, H., Yang, G., Zuo, X., Tang, H., Yang, Q., Li, G. Computational studies on the structural, electronic and optical properties of graphene-like MXenes (M_2CT_2, M = Ti, Zr, Hf; T = O, F, OH) and their potential applications as visible-light driven photocatalysts. *J. Mater. Chem. A* **4**, 12913-12920 (2016).
34. Zhang, H., Wu, D., Tang, Q., Liu, L., Zhou, Z. ZnO–GaN heterostructured

nanosheets for solar energy harvesting: computational studies based on hybrid density functional theory. *J. Mater. Chem. A* **1**, 2231-2237 (2013).

35. Xie, X., et al. Efficient photo-degradation of dyes using $CuWO_4$ nanoparticles with electron sacrificial agents: a combination of experimental and theoretical exploration. *RSC Advances* **6**, 953-959(2016).

36. Xiao, Y., Chen, M., Yang, Y., Zhang, M., & Huang, L. Ab initio research of a metal-free C/N van der Waals bilayer heterostructure with graphdiyne and g-C_3N_4. Jpn. *J. Appl. Phys.* **58**, 030906 (2019).
